# Detection of Chagas disease from the ECG: the George B. Moody PhysioNet Challenge 2025

**DOI:** 10.1088/1361-6579/aead44

**Published:** 2026-10-20

**Authors:** Matthew A Reyna, Soheil Saghafi, Zuzana Koscova, Audrey Weigle, Jan Pavlus, Andoni Elola, Salman Seyedi, Kiersten Campbell, Qiao Li, Sandhi M Barreto, Ester C Sabino, Antônio H Ribeiro, Antonio Luiz P Ribeiro, Reza Sameni, Gari D Clifford

**Affiliations:** 1 Department of Biomedical Informatics, Emory University, Atlanta, GA, United States of America; 2 Department of Electronic Technology, University of the Basque Country EHU, Bilbao, Spain; 3 Department of Preventive and Social Medicine, Faculdade de Medicina, Universidade Federal de Minas Gerais, Belo Horizonte, Brazil; 4 Instituto de Medicina Tropical da Faculdade de Medicina da Universidade de São Paulo, São Paulo, Brazil; 5 Department of Information Technology, Uppsala University, Uppsala, Sweden; 6 Science for Life Laboratory (SciLifeLab), Uppsala, Sweden; 7 Department of Internal Medicine, Faculdade de Medicina, Universidade Federal de Minas Gerais, Belo Horizonte, Brazil; 8 Telehealth Center and Cardiology Service, Hospital das Clínicas, Universidade Federal de Minas Gerais, Belo Horizonte, Brazil; 9 Department of Biomedical Engineering, Georgia Institute of Technology and Emory University, Atlanta, GA, United States of America; 10 Department of Biomedical Engineering, Johns Hopkins University, Baltimore, MD, United States of America; 11 Department of Anesthesiology and Critical Care Medicine, Johns Hopkins University, Baltimore, MD, United States of America

**Keywords:** electrocardiogram, Chagas disease, machine learning, PhysioNet challenge

## Abstract

*Objective.* Chagas disease is a parasitic infection that is endemic to Latin America and primarily transmitted by insects. When untreated, Chagas disease can cause cardiomyopathy. Serological testing capacities for Chagas disease are limited, but Chagas cardiomyopathy often manifests in electrocardiograms (ECGs), providing an opportunity to use ECG-based screening to prioritize patients for testing and treatment. *Approach.* The George B. Moody PhysioNet Challenge 2025 invited teams to develop algorithmic approaches for identifying potential cases of Chagas disease from ECGs. The Challenge metric, denoted by TPR@5%, measured the true positive rate of the top 5% of patients determined by an algorithm to be the most likely to have Chagas disease, reflecting the Brazilian serological testing capacity. The data included more than 360 000 ECGs from five Brazilian databases. We also developed an ensemble algorithm for the best-performing entries. *Main results.* This Challenge provided multiple innovations. First, we leveraged several datasets with labels from either patient reports or serological testing, providing a large dataset with weak labels and smaller datasets with strong labels. Second, we augmented the data to support model robustness and generalizability to unseen data sources. Third, we applied an evaluation metric, TPR@5%, that captured the local serological testing capacity for Chagas disease to frame the Challenge as a triage task. Fourth, we developed an ensemble model that combined the individual Challenge models to improve performance. These innovations provided reliable sensitivity at clinically relevant operating points. The performance of the top-performing Challenge model (Biomed-Cardio) varied performed well across datasets (TPR@5% of 0.468 on REDS-II and TPR@5% of 0.125 on ELSA-Brasil), and our ensemble-based approach demonstrated consistent performance across datasets (TPR@5% of 0.389 on REDS-II and TPR@5% of 0.146 on ELSA-Brasil). *Significance.* Over 630 participants from 111 teams submitted over 1300 entries during the Challenge, representing diverse approaches from academia and industry worldwide.

## 1. Introduction

The George B. Moody PhysioNet Challenges are annual competitions that support the development of open-source approaches to complex physiological and clinical problems (Goldberger *et al* 2000). For the PhysioNet Challenge 2025, we invited teams to develop algorithms for using electrocardiograms (ECGs) to identify potential cases of Chagas disease, helping to prioritize patients for confirmatory diagnosis and treatment.

Chagas disease is a tropical parasitic disease that is caused by protozoan *Trypanosoma cruzi* and primarily transmitted by triatomine bugs. It is endemic to South America, Central America, and Mexico. Migration has introduced Chagas disease to the United States and Europe, and local transmission has occurred the U.S. (Beatty *et al* 2025). Chagas disease affects more than 10.5 million people worldwide with 352 thousand annual infections and 8420 deaths in 2026 (Cousin *et al* 2026). There is no human vaccine for Chagas disease (Camargo *et al* 2022, Teixeira *et al* 2025).

After an acute phase, which frequently occurs in childhood, Chagas disease enters a life-long chronic phase (Nunes *et al* 2018, Sabino *et al* 2024). In the early stages of infection, Chagas disease has no or mild symptoms, and specific drugs can often cure the disease. In the later stages of infection, Chagas disease can cause cardiomyopathy, leading to heart failure, cardiac arrhythmias, and thromboembolism, increasing risks of death. Drugs are unlikely to cure the disease and cannot reverse the damage caused by the disease, but they may be able to arrest the progression of the disease. Serological testing has shown the widespread prevalence of Chagas disease in some areas, and such tests can be used for diagnosis in individual patients, but serological testing capacities are limited in endemic regions.

Given the individual and systemic harms of untreated cases of Chagas disease, improving the detection of Chagas disease promises to improve outcomes and lower costs. In many countries, detection rates are below 10%, or, more frequently, below 1%, preventing patients from receiving timely and effective treatment (World Health Organization 2022, Teixeira *et al* 2025). While serological testing is the gold standard for diagnosing Chagas disease, machine learning can help to prioritize potential Chagas disease patients for confirmatory testing with limited serological testing resources.

For example, Ghilardi *et al* (2024) showed that simple sociodemographic and environmental risk factors can improve detection, and physiological signals can provide additional clues. Since Chagas cardiomyopathy often manifests in ECGs, physiological symptoms can provide a complementary signal for Chagas disease and inform the treatment of Chagas-associated heart conditions (Ribeiro *et al* 2014).

ECG provides a widely available, low-cost, and non-invasive tool to capture the electrical activity of the heart. Early research demonstrated that alterations in heart rate variability (HRV) and ECG patterns are present even before overt cardiac involvement becomes clinically evident. For example, studies have reported changes in spectral indices of HRV among Chagas patients, indicating autonomic nervous system dysfunction (Vizcardo *et al* 2007, 2019). These early findings established HRV as a potential marker for identifying subclinical disease progression.

Approximate entropy and permutation entropy methods have been applied to Holter ECG recordings to discriminate between healthy individuals and Chagas patients, with evidence that these metrics can detect autonomic disturbances even in seropositive patients without overt ECG abnormalities (Cardoso *et al* 2015, Vizcardo and Ravelo Garcia 2018, Rodrigo Cornejo Luque *et al* 2020, Cornejo *et al* 2022). Such approaches suggested that nonlinear characterization may enhance early risk stratification, providing insights beyond conventional diagnostic tests (Vizcardo *et al* 2019). More recently, these entropy-based methods have been integrated with machine learning techniques to improve predictive performance (Isaac Valdez *et al* 2023, Rodriguez *et al* 2023).

Parallel to these methodological advances, the broader field of artificial intelligence has demonstrated transformative potential for automated ECG analysis. Deep learning models have achieved high performance in classifying common arrhythmias and conduction abnormalities, while also enabling the prediction of latent conditions not traditionally identifiable from the ECG alone. Within the context of Chagas disease, deep learning has been applied to predict left ventricular systolic dysfunction directly from the ECG in large patient cohorts (Brito *et al* 2021), and to develop screening models capable of distinguishing seropositive individuals using only standard ECG recordings (Jidling *et al* 2023). Complementary work in animal models further supports the potential of ECG-derived markers to differentiate between acute and chronic phases of *Trypanosoma cruzi* infection(Haro *et al* 2023).

These studies underscore both the promise and the challenges of leveraging ECGs for screening patients for Chagas disease. A particular influence on this work is the study by Jidling *et al* (2023), which illustrates both the remarkable potential of data-driven ECG analysis to improve disease detection and the substantial difficulties posed by performance drops across independent cohorts.

For the George B. Moody PhysioNet Challenge 2025, we sought to continue efforts to use ECGs to support screening for Chagas disease.

## 2. Methods

### 2.1. Challenge data

For the PhysioNet Challenge 2025, we assembled 12-lead ECG recordings and Chagas disease labels from several sources. We prepared these datasets for the Challenge to create a public training set and hidden validation and test sets for the Challenge.

We used 378 624 12-lead ECG recordings from 6 different sources. These sources of ECG data are described below and summarized in table 1.

**Table 1.** Patient-level summary of the data sources for the Challenge before data augmentation for the public training and hidden validation and test sets. Q1 and Q3 are the first and third quartiles, respectively.

Database	Age			Sex Female (%)	Recordings	Chagas Disease Prevalence
	Mean	Q1	Q3			
CODE-15%	53.2	37.0	70.0	59.7%	335,621	1.92%
SaMi-Trop	59.4	50.0	69.0	67.3%	1,631	100.00%
PTB-XL	62.8	50.0	72.0	47.9%	21,779	0.00%
REDS-II	57.1	50.0	65.0	50.7%	1,979	53.48%
SaMi-Trop 3	58.4	48.5	69.3	62.9%	3,854	28.50%
ELSA-Brasil	52.2	45.0	59.0	54.5%	13,739	2.04%

The Challenge data included 12-lead ECG data and Chagas disease labels from multiple public and private sources:

• The **CODE-15%** dataset (Ribeiro *et al* 2020) contains 345 779 ECG records from 233 770 patients from Brazil with self-reported Chagas disease status. Approximately 2% of patients reported positive cases of Chagas disease. The signals are approximately 10 s in length, and the sampling frequency is 400 Hz. This dataset is public and part of the Challenge training set.
• The **SaMi-Trop** dataset (Cardoso *et al* 2016) contains 1631 ECG records from 1959 patients from Brazil with Chagas cardiomyopathy. All patients were serologically validated for Chagas disease. The signals are approximately 10 s in length, and the sampling frequency is 400 Hz. This dataset is public and part of the Challenge training set.
• The **PTB-XL** database from the Physikalisch-Technische Bundesanstalt (PTB), Brunswick, Germany (Wagner *et al* 2020) contains 21 799 ECG records from 18 869 patients. All patients were assumed to be Chagas negative because of their geographical location in Europe, where Chagas disease is not endemic, but this assumption was not confirmed with serological testing. The signals are 10 s in length, and the sampling frequency is 500 Hz. This dataset is public and part of the Challenge training set.
• The **REDS-II** database (Nunes *et al* 2021) contains 1979 ECG records from 631 Brazilian patients with both positive and negative Chagas labels from serological testing. The dataset was constructed so that the numbers of Chagas positive and negative cases were approximately equal, so we oversampled the Chagas negative cases to create a positivity rate of 2%. The signals are approximately 10 s in length, and the sampling frequency is 300 Hz. This dataset is private and part of the Challenge validation and test sets.
• The **SaMi-Trop 3** database (Ribeiro *et al* 2026) contains 3855 ECG records from Brazilian patients with both positive and negative Chagas labels from serological testing. The dataset was constructed so that the numbers of Chagas positive and negative cases were approximately equal, so we oversampled the Chagas negative cases to create a positivity rate of 2%. The signals are approximately 10 s in length, and the sampling frequency varies, including 300 Hz, 500 Hz, 600 Hz, and 1000 Hz. This dataset is private and part of the Challenge test set.
• The **ELSA-Brasil** database (Pinto-Filho *et al* 2020) contains 13 739 ECG records from 13 739 Brazilian patients with both positive and negative Chagas labels from serological testing. Approximately 2% of patients tested positive for Chagas disease. The signals are approximately 10 s in length, and the sampling frequency is 300 Hz. This dataset is private and part of the Challenge test set.

### 2.2. Challenge data preparation

The different data sources, so we reformatted each record in a WFDB format so that data from different sources shared a consistent format. Some data sources zero-padded the ECG signals so that they could more easily store different records with different durations, so we removed samples at the beginning and end of a signal that had zero values for each channel of the ECG, and we removed records for signals that had zero values for each sample. Moreover, we replaced ages above 89 with a single age of 90 as needed to de-identify the data according to the Safe Harbor method (U.S. Department of Health and Human Services 2012).

#### 2.2.1. Challenge data source identification

Since the Challenge data sources had different prevalence rates, we sought to understand whether classifiers trained to distinguish Chagas-positive and Chagas-negative patients might inadvertently learn to distinguish different data sources based on regional or dataset-specific characteristics (i.e. ‘side features’) rather than the disease itself. To explore this possibility, we used power spectral density (PSD)-derived features to attempt classify the origin of each dataset independently of the Chagas disease labels. We normalized the power spectrum by first resampling each raw ECG lead to a uniform target frequency of 250 Hz to account for differences in sampling rates across records. Next, we standardized each ECG lead before using Welch’s method to compute its PSD. Specifically, we centered each lead by subtracting its mean and scaled it by dividing its standard deviation, ensuring that all leads had zero mean and unit variance. This normalization step removed amplitude differences caused by variations in electrode placement, signal gain, or subject physiology, allowing the resulting power spectra to reflect relative frequency content rather than absolute signal magnitude.

To characterize the spectral properties of ECG signals across datasets, we computed the average PSD of lead I using Welch’s method. Figure 1 displays the median PSD curves for randomly selected 1000 patients in each dataset, revealing distinct spectral signatures and highlighting dataset-specific noise characteristics, such as narrowband noise near 60 Hz and around 120 Hz. To quantify the relevance of different frequency bands for classification, we computed the average feature importance for each frequency component using a random forest model trained on spectral features. As shown in figure 2, peaks in feature importance correspond to frequencies near 60 Hz and 120 Hz, suggesting that these bands, which likely capture powerline interference, may contribute discriminative information unrelated to physiology or pathology such as Chagas disease. Additionally, the frequency bands 0–3 Hz, 50–70 Hz and 115–125 Hz emphasize their elevated importance across models. These findings informed the selection and interpretation of frequency-domain features in subsequent classification tasks. In this analysis, we estimated feature importance with a random forest model, which uses a non-linear, ensemble-based approach to evaluate how much each feature contributes to classification. Specifically, random forests consist of many decision trees, and each time a feature is used to split a node, the algorithm records how much that split reduces the classification impurity as measured via the Gini index. These reductions are averaged across all trees and normalized, producing a feature importance score that reflects the overall contribution of each input variable. Unlike linear models such as logistic regression, which rely on weighted coefficients, random forests capture non-linear relationships and interactions among features, making this importance measure particularly useful for complex, high-dimensional data such as spectral features. This approach follows the standard mean decrease in impurity method (Breiman 2001).

**Figure 1.**
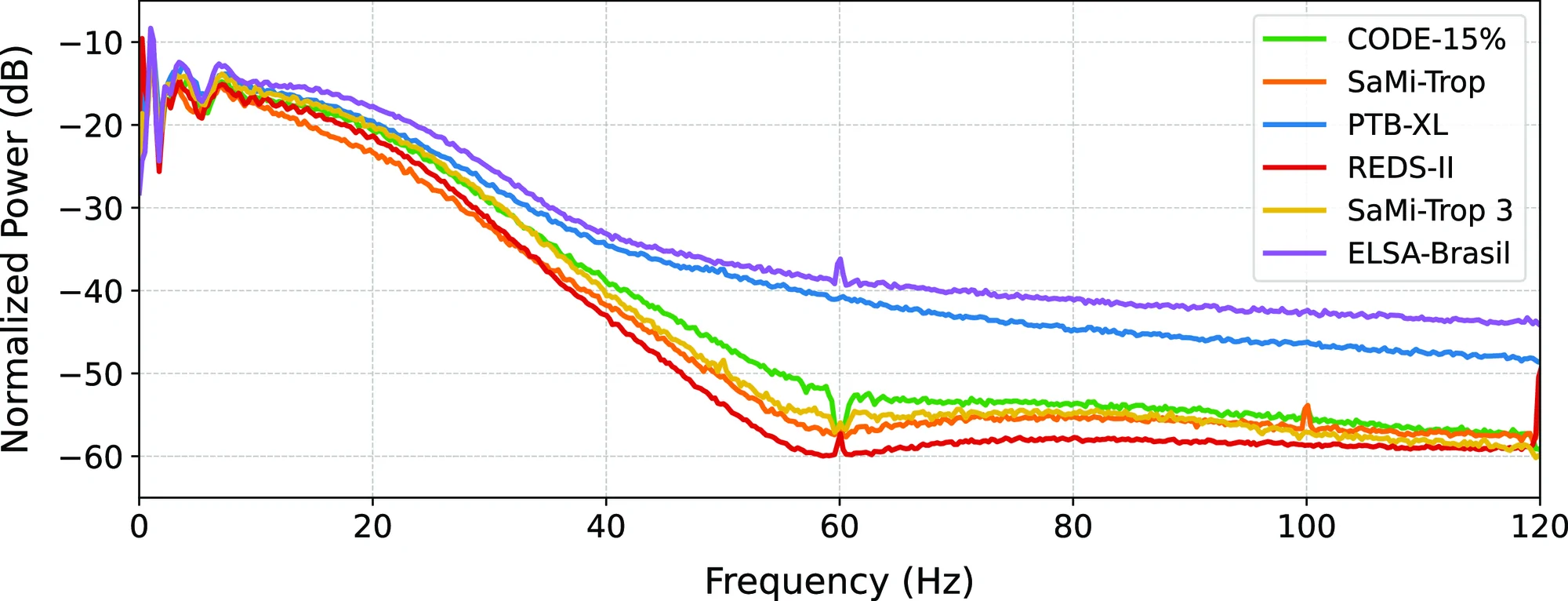
Median power spectral density of the first lead averaged across all 1000 patients for each dataset. Spectral estimates were computed using Welch’s method, with each curve representing the average profile per dataset. Peaks near 60 Hz and 120 Hz reflect powerline interference, while differences in spectral shape highlight variability in recording conditions and signal characteristics across datasets.

**Figure 2.**
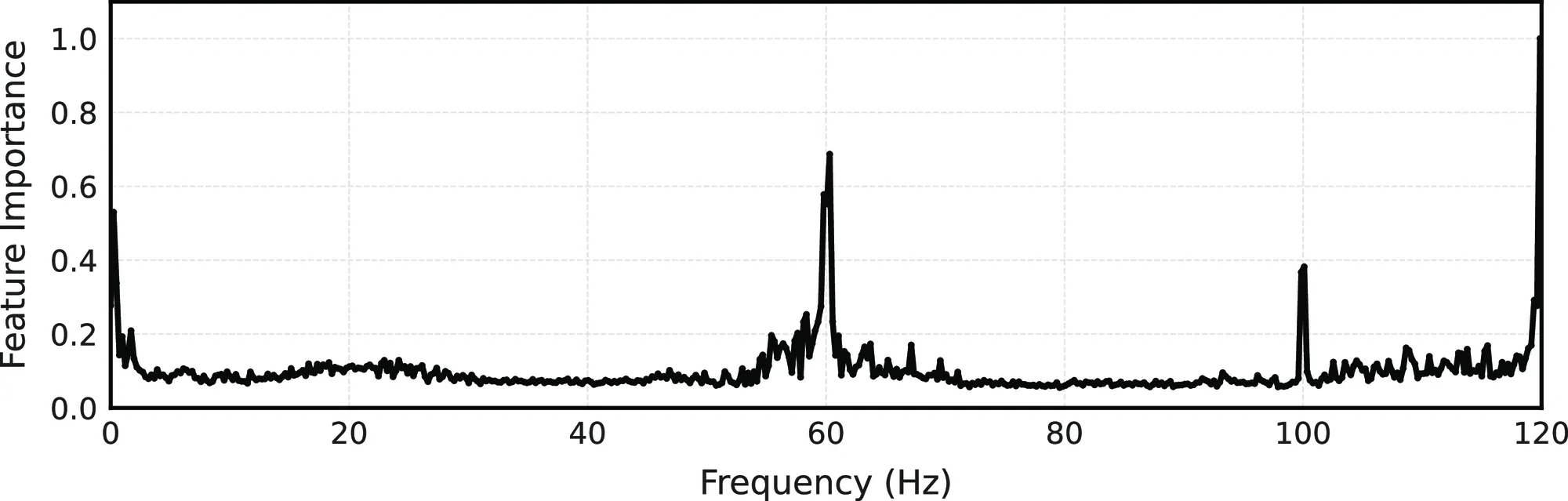
Feature importance across frequency bands, highlighting increased model reliance on the region near 60 Hz (the powerline frequency) during training.

The confusion matrix in figure 3 illustrates the performance of the dataset classification model across six ECG datasets, totaling 1200 ECGs: CODE-15% (200), SaMi-Trop (200), PTB-XL (200), REDS-II (200), SaMi-Trop 3 (200), and ELSA-Brasil (200). The model demonstrates a strong ability to distinguish between datasets based on their signal characteristics, indicating that the classifier successfully learned discriminative features unique to each dataset, despite potential overlap in clinical or recording contexts. Some confusion is observed between spectrally similar datasets, such as REDS-II and SaMi-Trop 3, suggesting shared patterns or hardware-related similarities. Overall, the results confirm that meaningful dataset-level distinctions exist in the spectral ECG features, which the classifier was able to capture effectively. Given that we do not want teams to use non-causal information (such as the association of powerline noise with class prevalence), it was important to develop a process to reduce these differences in the hidden validation and test sets.

**Figure 3.**
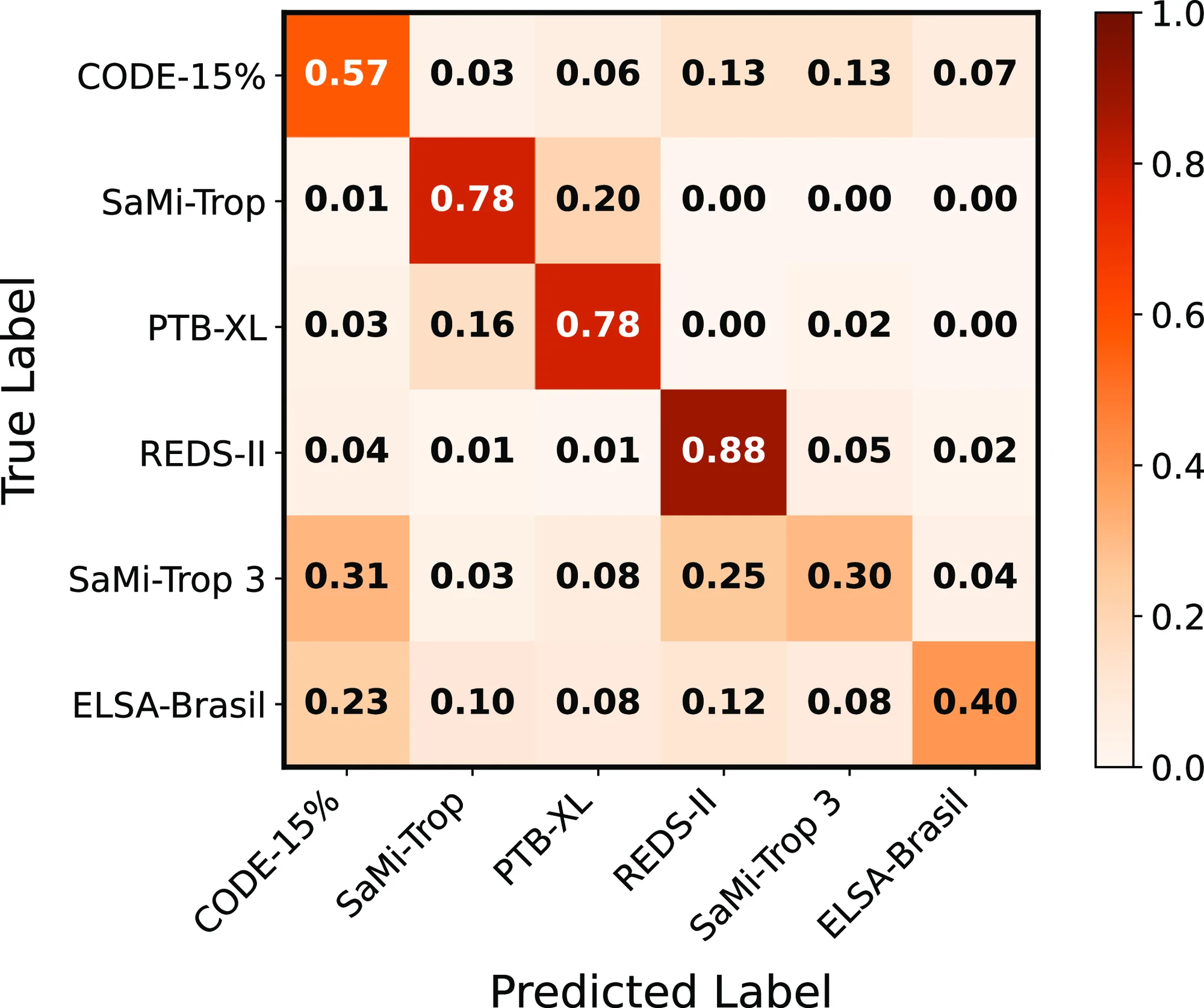
Normalized confusion matrix for dataset classification, where each cell represents the proportion of predictions normalized by the total number of samples in the corresponding true class (row).

#### 2.2.2. Challenge data augmentation

To help to address issue, we constructed the hidden validation and test sets so that each data source had similar prevalence rates of Chagas disease.

In particular, we oversampled the Chagas-negative records in the REDS-II and SaMi-Trop 3 datasets, which were collected with similar numbers of Chagas-positive and Chagas-negative patients, so that we could approximately match the prevalence rate of the ELSA-Brasil data, which has a 2.04% positivity rate.

For both Chagas-positive and Chagas-negative records in the validation and test sets, we also applied the following transformations:

1. To support the robustness of the models to different storage formats, we changed the analog-to-digital (ADC) gains of the digital signals, chosen uniformly at random from 0.4, 0.8, 1, 1.25, and 2.5, while preserving the physical values of the signals.
2. To support the robustness of the models to different storage formats, we changed the ADC baselines of the digital signals, chosen uniformly at random from −0.1, −0.05, −0.025, 0, 0.025, 0.05, and 0.1, while preserving the physical values of the signals.
3. To support the robustness of the models to noise, we added a source of noise, chosen uniformly at random from the baseline wander, electrode motion, and muscle artifact signals in Moody *et al* (1992), with a target signal-to-noise ratio (chosen uniformly at random from 40 dB, 45 dB, and 50 dB).
4. To support the robustness of the models to different device filters, we applied a filter coefficient, chosen uniformly at random from 40–55 Hz, 60–75 Hz, and 80–90 Hz.
5. To support the robustness of the models to different device sampling frequencies, a new sampling frequency, chosen uniformly at random from 300 Hz, 400 Hz, 500 Hz, 600 Hz, 700 Hz, 800 Hz, 900 Hz, 1000 Hz.
6. To support robustness to different sources and formats, we removed the source information from the records and renamed the records at random.

These steps varied the digital representations of the data, added small amounts of noise, applied different filters, resampled the data to different sampling frequencies to create new but highly similar records. The number of recordings and the prevalence of Chagas disease in each database after data augmentation are shown in table 2.

**Table 2.** Patient-level summary of Challenge data after data augmentation for the public training and hidden validation and test sets. Q1 and Q3 are the first and third quartiles, respectively.

Database	Cohort	Age			Sex female (%)	Recordings	Chagas disease prevalence
		Mean	Q1	Q3			
CODE-15%	Training	53.2	37.0	70.0	59.7%	335,621	1.92%
SaMi-Trop	Training	59.4	50.0	69.0	67.3%	1,631	100.00%
PTB-XL	Training	62.8	50.0	72.0	47.9%	21,779	0.00%
REDS-II	Validation	58.5	52.0	66.0	53.1%	37,779	2.05%
REDS-II	Test	58.0	51.0	66.0	43.4%	13,720	2.04%
SaMi-Trop 3	Test	56.4	45.0	68.0	62.6%	53,444	2.06%
ELSA-Brasil	Test	52.2	45.0	59.0	54.5%	13,739	2.04%

### 2.3. Challenge objective

We asked participants to design and implement working, open-source algorithms for identifying cases of Chagas disease from standard 12-lead ECG recordings, helping to prioritize potential Chagas patients for confirmatory diagnosis and treatment. We required teams to submit code both for their trained models and for training their models, which aided the generalizability and reproducibility of the research conducted during the Challenge. We trained the participants’ models on the public training set and evaluated the resulting models on the hidden validation and test sets using a tailored evaluation metric that we designed for this year’s Challenge to reflect the confirmatory testing capacity of Chagas-endemic regions.

### 2.4. Challenge overview, rules, and expectations

This year’s Challenge was the 26th George B. Moody PhysioNet Challenge (Goldberger *et al* 2000). As in previous years, the Challenge had an unofficial phase and an official phase. The unofficial phase (9 January to 9 April 2025) introduced the teams to the Challenge and provided an opportunity to discuss the topic with and seek feedback from the teams about the data, evaluation metrics, and evaluation environment. For the unofficial phase, we publicly shared the Challenge objective, training data, example algorithms, and evaluation metric and invited the teams to submit their code for evaluation on the validation set, scoring at most five entries from each team on the validation set. Between the unofficial and official phases, we took a hiatus (10 April to 28 May 2025) to improve the Challenge. The official phase (29 May to 20 August 2025) continued the Challenge and provided an opportunity for teams to refine their methods. For the official phase, we updated the Challenge data, example algorithms, and evaluation metric and again invited teams to submit their code for evaluation, scoring at most ten entries from each team on the validation set. After the official phase, we evaluated a single entry from each team on the test set. Moreover, while teams were encouraged to ask questions, pose concerns, and discuss the Challenge in a public forum, we prohibited them from discussing their particular approaches to preserve the uniqueness of their approaches to solving the problem posed by the Challenge.

When evaluating the submissions, we first ran each team’s training code on the training data and then ran each team’s trained model from the previous step on the hidden data. To better capture the clinical environments in which an algorithm may be deployed, we ran each algorithm sequentially on the recordings.

We allowed teams to submit either MATLAB or Python implementations of their code. Other programming languages were supported by request, but no teams requested another programming language. Participants containerized their code in Docker-compatible environments, which allowed them to more explicitly describe the runtime environments for their code, and they shared them with us using git-compatible repositories. We downloaded and ran their containerized code. The computational environment is described more fully in Reyna *et al* (2020).

We used a containerized environment with 16 vCPUs, 60 GB RAM, at least 100 GB of available local storage, and an optional GPU, which was either an Ampere A30 GPU or an RTX 6000 Ada Generation GPU, depending on the available resources as the time of running the entry.

We imposed a 72 h time limit on training with a GPU, a 96 h time limit on training without a GPU, and a 48 h time limit on inference for each of the validation or test sets.

To aid teams, we shared example models that we implemented in MATLAB and Python. Both the MATLAB and Python example models were random forest models that used the mean and standard deviation of the values in each channel of each signal as well as the available demographic information. These example models were not designed to be competitive but instead to provide minimal working examples of how to read the data and how to return the results for evaluation.

#### 2.4.1. Challenge scoring metric

In many Chagas-endemic regions, the availability of serological testing is severely limited due to financial, logistical, and infrastructural constraints. As a result, even if an automated system flags many patients as potentially positive based on their ECGs, only a small proportion of the flagged patients can realistically be referred for confirmatory testing. This mismatch between diagnostic need and testing capacity fundamentally alters the design requirements for machine learning systems deployed in such settings.

To reflect this constraint, the Challenge evaluated algorithms based on their ability to *prioritize true Chagas-positive patients within a fixed referral capacity*, rather than on traditional machine learning metrics such as the area under the receiver-operating characteristic (AUROC) curve or accuracy. Specifically, we scored each algorithm by computing the *true positive rate (TPR) among the top 5% of patients ranked by a model, denoted by TPR@5%*. The 5% quantity corresponds to an estimated bound for the testing capacity in many real-world scenarios in Brazil: *how many Chagas-positive patients can an algorithm prioritize for confirmatory testing with a constrained testing capacity?*

Formally, let T=P+N denote the total number of subjects in the study, where P and N represent the number of Chagas-positive and Chagas-negative cases, respectively. Also, let M denote the maximum number of subjects that can be referred for serological testing, regardless of the eventual outcome of testing. Next, suppose that each subject is assigned a risk score by a classifier, and those with scores exceeding a threshold τ are classified as positive (i.e. referred for testing). We define the *TPR* and the *false positive rate* (FPR) at threshold τ as $\mathrm{tpr}(\tau) = \mathrm{TP}(\tau)/P$ and $\mathrm{fpr}(\tau) = \mathrm{FP}(\tau)/N$, where $\mathrm{TP}(\tau)$ and $\mathrm{FP}(\tau)$ are the number of true positives and false positives, respectively, at a threshold τ.

To ensure the classifier operates within the referral constraint, we require that the total number of referred subjects not exceed M, i.e. $\mathrm{TP}(\tau) + \mathrm{FP}(\tau) \,{\unicode{x2A7D}}\, M$, or:

\begin{align*} \pi_p \cdot \mathrm{tpr}\left(\tau\right) + \pi_n \cdot \mathrm{fpr}\left(\tau\right) \,{\unicode{x2A7D}}\, m,\end{align*}

where $\pi_p : = P/T$ and $\pi_n : = N/T = 1 - \pi_p$ are the class proportions for positive and negative cases, respectively, and the ratio m:=M/T represents the *maximum admission rate* (cf Sameni 2025 for further details). Equation (1) defines a feasible region in ROC space bounded by a line with slope $-\pi_n/\pi_p = -(1-\pi_p)/\pi_p$, intersecting the TPR axis at $m/\pi_p = M/P$ and the FPR axis at $m/\pi_n = M/N$, as illustrated in figure 4. The optimal operating point of a classifier under this constraint lies at the intersection of its receiver-operating characteristic (ROC) curve with this boundary, i.e. where a given classifier detects its maximum number of true positive cases, within the testing capacity.

**Figure 4.**
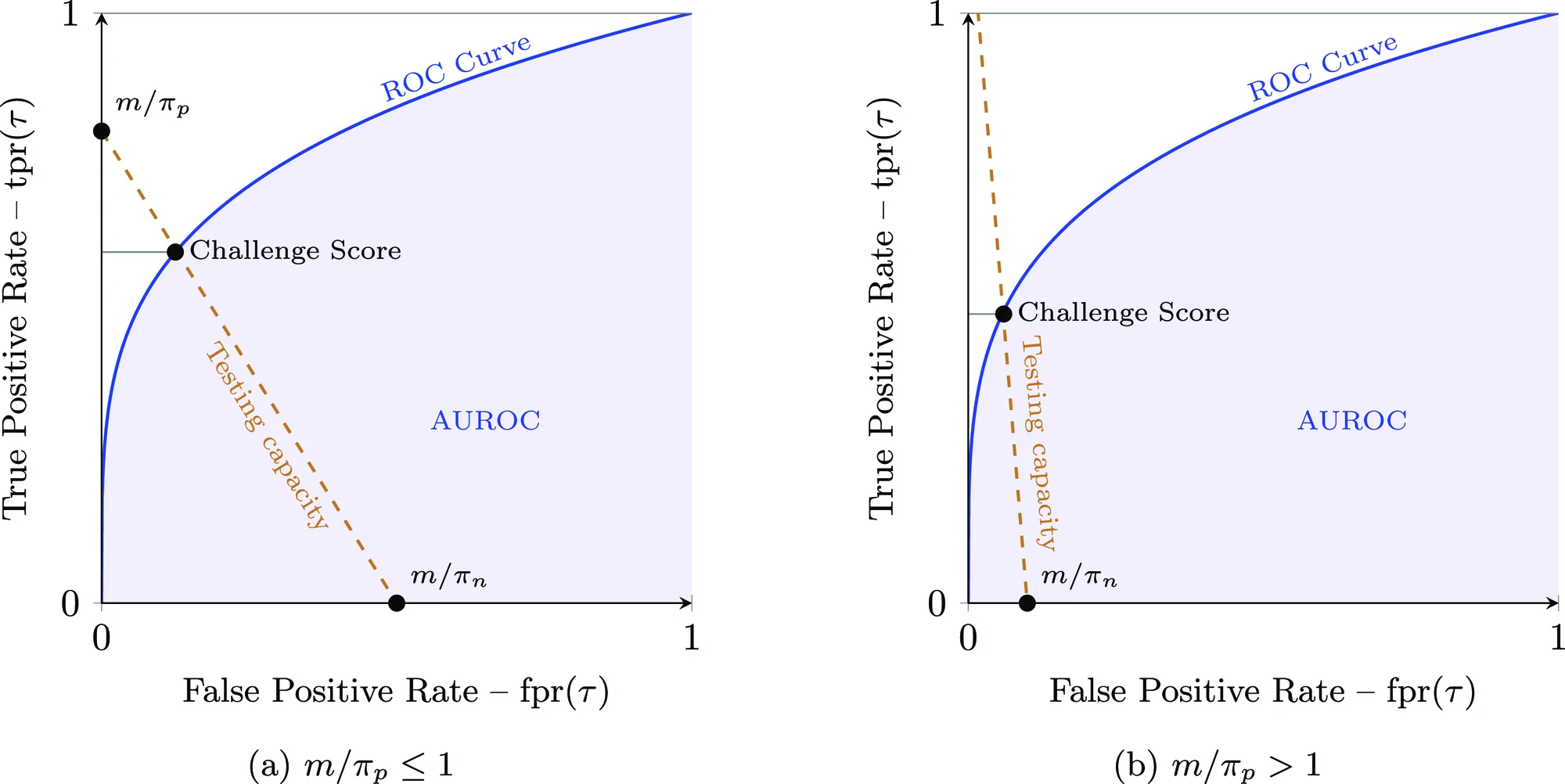
Illustration of the Challenge evaluation metric in receiver-operating characteristic (ROC) space. The brown dashed lines denotes the loci of operating points corresponding to a fixed total testing capacity (i.e. the total number of cases predicted as positive, regardless of whether they are true or false positives). See Sameni (2025) for details and the geometric interpretation. The left panel corresponds to $m/\pi_p \,{\unicode{x2A7D}}\, 1$, where the maximum testing capacity is smaller than the actual number of positive cases, while the right panel corresponds to $m/\pi_p > 1$, where the testing capacity exceeds the number of positives. The Challenge dataset falls into the latter category ($m/\pi_p > 1$). The triangular region below the dashed-line boundaries represents the feasible operating region under the fixed testing-capacity constraint. The Challenge score, TPR@5%, is defined as the TPR achieved within the top m=5% of predicted cases, approximating a practical limit on serological testing capacity. Across both scenarios, the maximum achievable Challenge score for an ideal classifier is $\min(1, m/\pi_p)$, which equals 1 for the Challenge dataset considered here.

Rather than requiring participants to tune their threshold τ explicitly, we evaluated each algorithm’s ability to identify Chagas-positive cases within the top M ranked predictions. If multiple cases received the same risk score so that no threshold τ corresponded to exactly M cases, then we returned the expected number of positive cases for reordering the tied cases at random.

This formulation reframes the task as a constrained ranking problem, closely reflecting deployment conditions where serological testing is scarce. The metric prioritizes high-precision triage and encourages models that are both discriminative and resource-aware which are essential characteristics for scalable Chagas disease screening. As illustrated in figure 4, the Challenge score is related to popular metrics such as the AUROC, but it is different in meaningful ways. An ideal classifier with an AUROC of 1 would achieve a perfect Challenge score; however, among practical classifiers, higher scores are awarded to models with stronger performance (higher TPR) near the operating point where the testing-capacity constraint line intersects the model ROC curve. During the Challenge, the teams were informed of the 5% testing capacity, and an approximate 2%–3% Chagas prevalence across datasets was communicated to them in the Challenge Forum discussions; however, the exact prevalence of the hidden test set was not provided to avoid overfitting and preserve generalizability. Therefore, the optimal strategy for classifier design and optimization would be to maximize performance (TPR or partial AUROC) near the operating point on the ROC curve, as determined by real-world Chagas disease prevalence and testing-capacity constraints.

### 2.5. Voting algorithms

To improve predictive performance and enhance clinical applicability, we also derived ensemble voting algorithms from the Challenge models. Ensemble learning combines predictions from multiple models with the goal of improving robustness, reducing variance, and mitigating model-specific biases. This approach is particularly useful in ECG-based disease detection, where individual models may capture different aspects of signal variability and noise.

For this approach, we defined three simple ensemble models using the arithmetic mean, geometric mean, and median of the model outputs for each record across the ten approaches with the highest performance on the test set. The ten models were selected according to the official Challenge score as an objective and reproducible criterion rather than through explicit optimization of ensemble diversity.

Although deep learning was common among the selected entries, the ten models represented heterogeneous modeling strategies. Six used CNN-based architectures spanning ResNet, U-Net with squeeze-and-excitation blocks, InceptionTime, SEResNet/ConvNeXt-style architectures, and a lightweight VGG-based design; three used Transformer or pretrained foundation-model architectures; and one used a CNN-BiLSTM-attention hybrid. The approaches also differed in their use of pretraining and transfer learning, demographic-data fusion, domain adaptation, knowledge distillation, recurrent modeling, and internal model ensembling. Thus, the selected models were not based on a single homogeneous architecture or feature-extraction strategy.

Nevertheless, architectural similarity alone does not necessarily imply similarity in learned representations or predictions, since independently developed models with similar high-level architectures may differ substantially in preprocessing, initialization, optimization, pretraining, training strategy, and postprocessing. The present analysis was therefore intended as a proof-of-concept aggregation of the highest-performing Challenge entries rather than an explicitly diversity-optimized ensemble. Diversity-aware model selection or optimized ensemble weighting based on prediction complementarity could be investigated in future work.

### 2.6. Baseline model

Jidling *et al* (2023) is an ensemble of 15 one-dimensional residual convolutional neural networks (ResNets) for detecting Chagas disease directly from 12-lead ECGs. Trained on the CODE-15% (Ribeiro *et al* 2020) and SaMi-Trop datasets (Cardoso *et al* 2016), the ensemble architecture combines predictions from multiple independently initialized networks and recognizes electrophysiological abnormalities associated with the disease, including patterns related to conduction disturbances and chronic Chagas cardiomyopathy. In this study, this model served as a baseline deep learning approach.

### 2.7. ECGFounder model

ECGFounder (Li *et al* 2025a) is a foundation model for 12-lead ECG analysis that was developed for detecting cardiac rhythms, conduction abnormalities, and other cardiovascular conditions, but not specifically for Chagas disease detection. Trained on the Harvard-Emory Database (Koscova *et al* 2026), ECGFounder was developed to learn general representations of ECG signals and can subsequently be adapted to a variety of downstream clinical prediction tasks. Given a raw 12-lead ECG recording, ECGFounder generates a 150-dimensional output vector corresponding to a fixed set of predefined ECG diagnostic categories, including cardiac rhythms, conduction abnormalities, and other electrocardiographic findings. Each dimension represents the model output score associated with a specific diagnostic category. The complete set of 150 diagnostic labels is defined in the supplementary material of Li *et al* (2025a). In this study, ECGFounder was used as a frozen feature extractor, with no fine-tuning or updating of its pretrained model parameters on the Challenge data. The resulting 150 diagnostic output scores generated for each ECG recording were subsequently used as the input feature set for a CatBoost classifier (Prokhorenkova *et al* 2018) to predict Chagas disease.

### 2.8. Feature importance

To improve model interpretability and enhance clinical applicability, we also performed feature importance analysis on the ECGFounder-derived feature representations. Feature importance methods (Lundberg and Lee 2017) quantify the contribution of individual features to model predictions, enabling identification of the ECG-derived representations that are most informative for Chagas disease detection. This approach is particularly useful in foundation-model-based ECG analysis, where latent features may capture complex electrophysiological patterns that are not readily apparent from raw ECG signals. By ranking features according to their predictive importance, we sought to better understand the factors driving model decisions and identify ECG characteristics potentially associated with known manifestations of Chagas cardiomyopathy. In this study, we used SHAP (SHapley Additive exPlanations) values (Lundberg and Lee 2017) to assess feature importance. Specifically, for each ECG recording, we used the 150-dimensional prediction vector generated by the ECGFounder model together with the corresponding Chagas disease label. A CatBoost classifier (Prokhorenkova *et al* 2018) was then trained using these features, and SHAP analysis was applied to quantify the contribution of each feature to the model’s predictions. This approach enabled us to identify the most influential ECGFounder-derived features associated with Chagas disease classification.

## 3. Results

We received a total of 1317 code submissions from 111 teams with over 650 team members during the course of the Challenge. There were 451 submissions during the unofficial phase, including 185 successful submissions and 266 unsuccessful submissions with 75 submissions in the last 24 hours of the unofficial phase. There were 866 submissions during the official phase, including 372 successful submissions and 494 unsuccessful submissions with 99 submissions in the last 24 h of the official phase.

After the end of the official phase, we attempted to score one entry from each team on the test set in section 2.1. A total of 65 teams had at least one entry that we could train on the public training set and evaluate on the hidden validation and test sets; a total of 40 teams met all of the requirements to be ranked, including an accepted Computing in Cardiology (CinC) 2025 conference abstract, a preprint and final CinC conference proceedings paper submission, and CinC conference registration.

Tables 3 and 4 summarize the team performance on the hidden validation and test sets; table 3 includes results for teams that met all of the requirements to be ranked, and table 4 includes results for teams that did not meet all of the requirements to be ranked. Team summaries, additional scores, and the full Challenge criteria for rankings are available in (Reyna *et al* 2025).

**Table 3.** Challenge scores on the validation set, which contains data from the REDS-II dataset, and the test set, which contains data from the REDS-II dataset, the SaMi-Trop 3 dataset, and the ELSA-Brasil dataset, for the ranked Challenge teams on the mean score across the three data sources in the test set. Teams are sorted by rank on the mean score across the three data sources in the test set.

Rank	Team name (CinC Submission ID)	REDS-II (validation)	REDS-II (test)	SaMi-Trop 3 (test)	ELSA-Brasil (test)	Mean test set
1	Biomed-Cardio (19) (Van Santvliet *et al* 2025)	0.445	0.468	0.376	0.125	0.323
2	DlaskaLabMUI (236) (Nicolson *et al* 2025)	0.440	0.357	0.375	0.118	0.283
3	AIChagas (100) (González Vázquez *et al* 2025)	0.360	0.382	0.329	0.129	0.280
4	ISIBrno-AIMT (132) (Nejedly *et al* 2025)	0.458	0.354	0.342	0.118	0.271
5	Ahus AIM (298) (Stenhede and Ranjbar 2025)	0.412	0.389	0.325	0.093	0.269
6	Cardio_Basel25 (42) (Champetier and Mueller 2025)	0.382	0.289	0.355	0.125	0.256
7	CinCo Amigos (158) (Wang *et al* 2025a)	0.338	0.339	0.247	0.164	0.250
8	Chagas_detector (416) (Nguyen *et al* 2025)	0.323	0.329	0.281	0.125	0.245
9	AIMED (368) (Soares *et al* 2025)	0.372	0.321	0.295	0.104	0.240
10	Chagas_UBT (126) (Mamaghani *et al* 2025)	0.343	0.314	0.295	0.104	0.238
10	MIWEAR (477) (Chen *et al* 2025)	0.379	0.282	0.308	0.125	0.238
12	Cha-Cha-Chagas (179) (Singstad *et al* 2025)	0.316	0.296	0.247	0.136	0.226
12	SwissBeatsNet (160) (Erlacher *et al* 2025)	0.425	0.286	0.320	0.071	0.226
14	Bug Busters (250) (Julius Sandelin *et al* 2025)	0.369	0.271	0.319	0.082	0.224
15	Med_YNNU (101) (Huang *et al* 2025)	0.299	0.304	0.276	0.082	0.221
16	PhysioWinn (86) (Dharia *et al* 2025)	0.326	0.311	0.253	0.096	0.220
17	CAUETUMN (475) (Im *et al* 2025)	0.347	0.282	0.272	0.100	0.218
18	ElectroDoChagas (285) (Sanchez *et al* 2025)	0.351	0.268	0.278	0.104	0.217
18	Leicester Fox (144) (Qaqos *et al* 2025)	0.258	0.350	0.259	0.043	0.217
18	Seoul Mates (219) (Kim 2025)	0.306	0.293	0.246	0.111	0.217
21	Medics (11) (Patidar 2025)	0.266	0.289	0.256	0.071	0.205
22	HeartGoesOut2U (197) (Kisieliūtė and Kolupayev 2025)	0.372	0.232	0.275	0.104	0.204
23	ChagAI (108) (Aublin *et al* 2025)	0.259	0.279	0.217	0.111	0.202
24	DataNinyas (234) (Bisgin *et al* 2025)	0.303	0.275	0.233	0.089	0.199
25	CLECLINIC (408) (Saini and Araujo 2025)	0.271	0.246	0.277	0.071	0.198
26	Kust_MeAI (237) (Zhu *et al* 2025)	0.201	0.254	0.231	0.100	0.195
27	UMC Utrecht (332) (B.S. Schots *et al* 2025)	0.280	0.314	0.227	0.036	0.192
29	ECGenius (283) (Shen *et al* 2025)	0.277	0.296	0.173	0.100	0.190
30	EPBandoleroLab (239) (Pascual-Mellado *et al* 2025)	0.259	0.161	0.224	0.136	0.174
31	FjordNet (91) (Dong *et al* 2025)	0.270	0.246	0.173	0.100	0.173
32	Revenger (309) (Wen and Kang 2025)	0.245	0.229	0.148	0.111	0.163
33	WEAIT (35) (Puszkarski *et al* 2025)	0.237	0.196	0.161	0.121	0.159
34	DEbuggers (37) (Haidamous *et al* 2025)	0.350	0.279	0.050	0.104	0.144
35	CompleXformers (61) (Clemente *et al* 2025)	0.187	0.149	0.116	0.093	0.119
36	Blue and Gold (192) (Dupont and Povinelli 2025)	0.150	0.139	0.125	0.071	0.112
37	SambaRhythm (259) (Long *et al* 2025)	0.099	0.089	0.121	0.114	0.108
38	GAIN-ECG (80) (Li *et al* 2025b)	0.090	0.089	0.095	0.061	0.082
39	Chaguys (335) (Taconne *et al* 2025)	0.088	0.086	0.091	0.050	0.076
40	CHA-CruzControl (243) (Herzler *et al* 2025)	0.050	0.050	0.050	0.050	0.050

**Table 4.** Challenge scores on the validation set, which contains data from the REDS-II dataset, and the test set, which contains data from the REDS-II dataset, the SaMi-Trop 3 dataset, and the ELSA-Brasil dataset, for the unranked Challenge teams on the mean score across the three datasets in the test set. Teams are sorted alphabetically by team name.

Team name (CinC Submission ID)	REDS-II (validation)	REDS-II (test)	SaMi-Trop 3 (test)	ELSA-Brasil (test)	Mean test set
ADI_KOS (308)	0.062	0.046	0.056	0.058	0.053
BAPORLab (235)	0.369	0.368	0.308	0.100	0.259
BuckeyeAI (447)	0.200	0.193	0.221	0.061	0.158
CardioAI (442) (Singh *et al* 2025)	0.245	0.268	0.219	0.093	0.193
CeZIS (238)	0.386	0.279	0.324	0.118	0.240
DeepTryp (279)	0.250	0.207	0.211	0.121	0.180
DLU (110)	0.343	0.300	0.238	0.107	0.215
DTU_HealthTech (129)	0.252	0.218	0.236	0.136	0.197
ECG_ChD_25 (77) (Li 2025)	0.372	0.282	0.232	0.086	0.200
ECG_UvA	0.115	0.193	0.107	0.071	0.124
ECGWizards	0.150	0.196	0.172	0.111	0.160
Ephemeris Labs (99) (Boysen *et al* 2025)	0.050	0.050	0.050	0.050	0.050
GDMUBME_ECGGroup (32)	0.311	0.289	0.305	0.121	0.238
Haarcells (449)	0.279	0.300	0.240	0.075	0.205
HAI_AMRITIANS (322)	0.231	0.189	0.183	0.100	0.157
IMP (34)	0.195	0.211	0.191	0.093	0.165
KHU_BME (27)	0.094	0.104	0.115	0.093	0.104
Logia_DC_UFC (224)	0.106	0.132	0.141	0.089	0.121
Mainchagas (48)	0.163	0.232	0.150	0.089	0.157
MCV_UVIC (428) (Jha and Mbilinyi 2025)	0.272	0.246	0.236	0.093	0.192
medex (51)	0.283	0.279	0.226	0.136	0.214
OpenHeart (315) (Ikurumi 2025)	0.101	0.064	0.066	0.039	0.056
OSUAI	0.328	0.268	0.295	0.107	0.223
PFAP (191) (Woo Lee and An 2025)	0.317	0.304	0.262	0.050	0.205
SZTU-Chagas	0.089	0.043	0.051	0.064	0.053
Ulmer	0.315	0.279	0.263	0.107	0.216

Figure 5 shows the performance of each team’s chosen entry on the validation and test sets. The median Challenge score dropped 1.4% (from 0.279 to 0.275) from the REDS-II data in the validation set to the REDS-II data in the test set, providing almost unchanged results. The median Challenge score dropped 15% (from 0.279 to 0.236) from the REDS-II data in the validation set to the SaMi-Trop 3 data in the test set. The median Challenge score dropped 64% (from 0.279 to 0.100) from the REDS-II data in the validation set to the ELSA-Brasil data in the test set.

**Figure 5.**
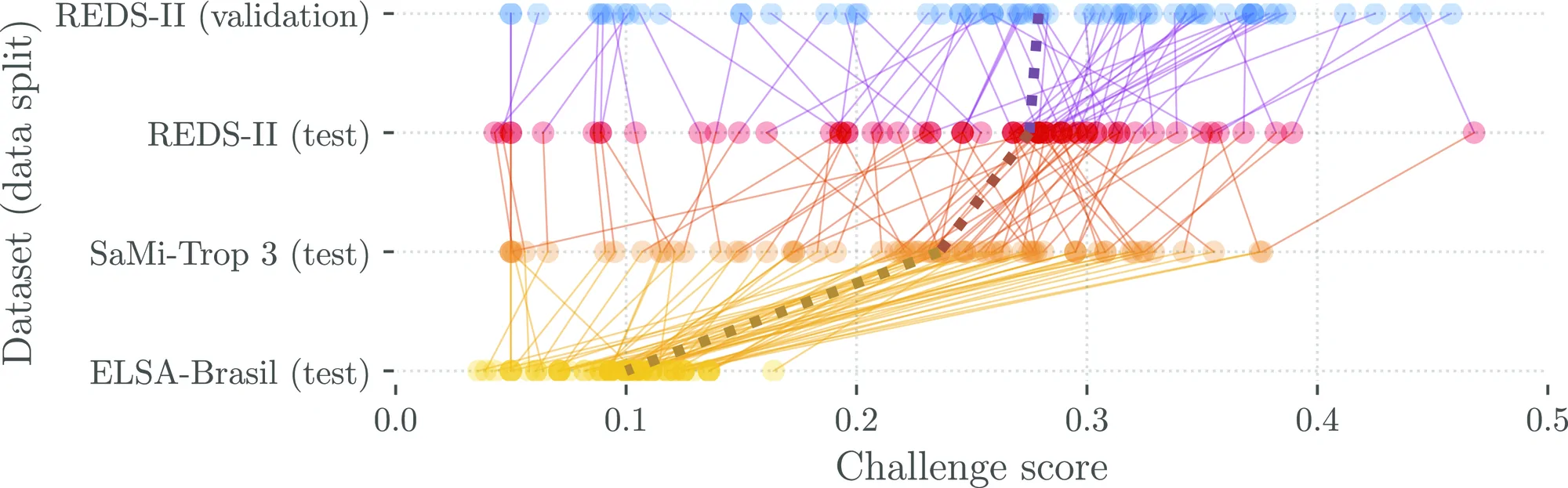
Challenge scores (x-axis) on the data sources (y-axis) for the hidden validation and test sets. Each point is the score of the method on a different dataset, and each thin solid line connects the scores for each team across datasets; the thick dashed color line shows the change in the median score across the different datasets.

Figure 6 shows the ROC curves for the top-performing individual PhysioNet Challenge models, the best-performing ensemble approach (arithmetic mean), a previously validated baseline model comprising an ensemble of 15 one-dimensional ResNet trained on large Brazilian ECG cohorts to identify Chagas disease directly from raw 12-lead ECG recordings (Jidling *et al* 2023), and the ECGFounder foundation model, which extracts 150-dimensional ECG feature embeddings from pretrained representations learned on large-scale ECG data (Li *et al* 2025a) across the three test datasets: REDS-II, SaMi-Trop 3, and ELSA-Brasil.

**Figure 6.**
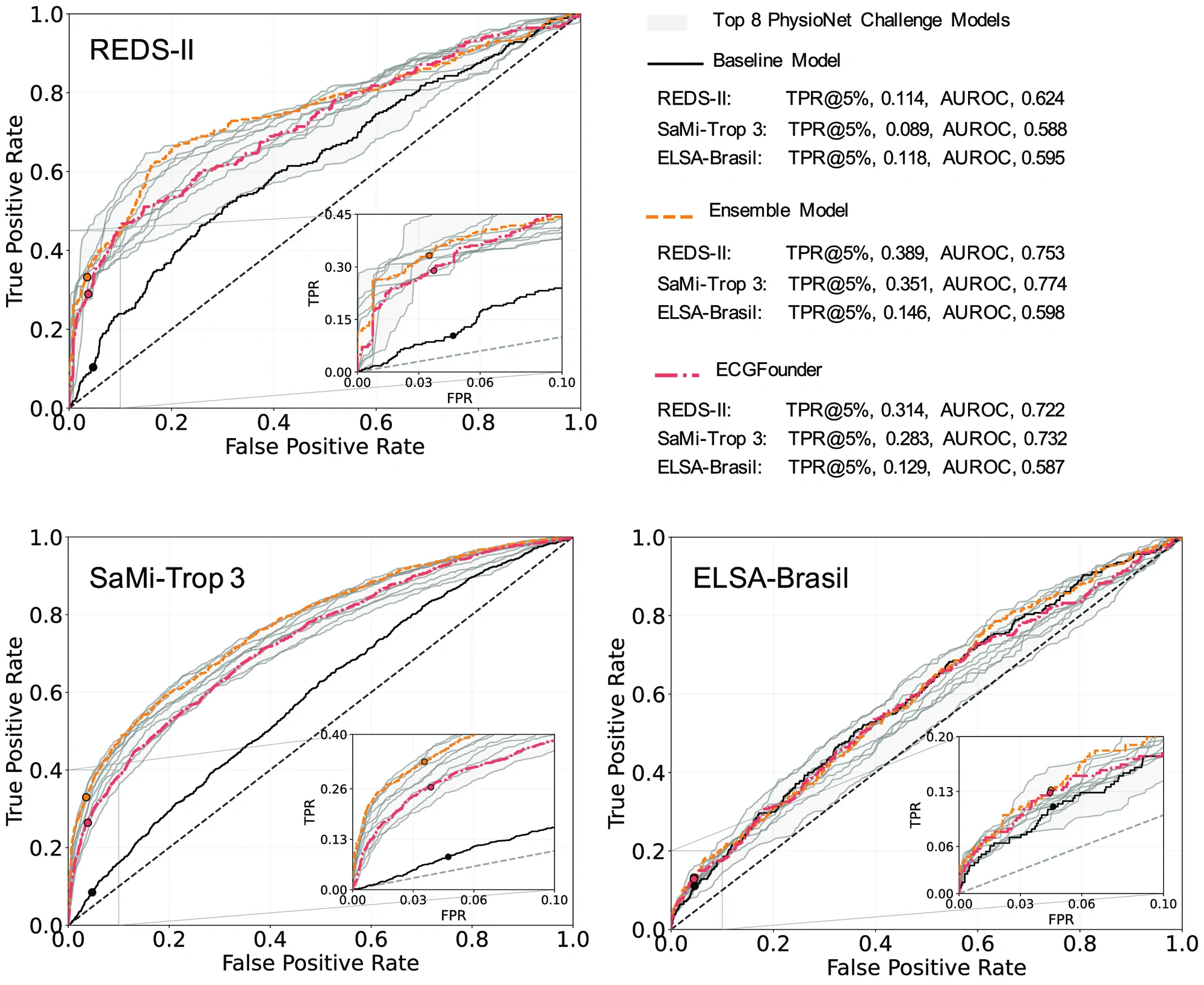
ROC curves comparing the baseline model, the top-performing individual PhysioNet Challenge models, the ECGFounder foundation model, and the best-performing ensemble (geometric mean) across the REDS-II, SaMi-Trop 3, and ELSA-Brasil datasets. The black curve represents the baseline model, while the gray shaded region captures the range of performance across the top eight individual models. The geometric mean ensemble (orange dashed curve) achieves the strongest overall performance on REDS-II and SaMi-Trop 3, with higher AUROC and TPR@5% values compared with both the ECGFounder foundation model and the baseline model. On ELSA-Brasil, performance differences across methods are smaller. Colored markers indicate operating points selected under a constrained maximum fraction of predicted positives. The insets highlight the low FPR region (FPR $\,{\unicode{x2A7D}}\, 0.1$), demonstrating the improved sensitivity and robustness of the ensemble approach in clinically relevant operating ranges.

Among the individual Challenge models, Biomed-Cardio achieves the highest average TPR@5% score (0.323), driven primarily by strong performance on REDS-II (0.468) and SaMi-Trop 3 (0.376). However, its performance decreases substantially on ELSA-Brasil (0.125), and was beaten by several other teams, including the ensemble models. In comparison, the arithmetic mean ensemble model demonstrates more stable performance across cohorts, suggesting improved robustness and generalizability through aggregation of complementary model predictions.

Across the datasets, the ensemble approach shows its strongest discriminative performance on REDS-II, followed by SaMi-Trop 3, with more modest performance on ELSA-Brasil. Specifically, the arithmetic mean ensemble achieves AUROC values of 0.753, 0.774, and 0.598 on REDS-II, SaMi-Trop 3, and ELSA-Brasil, respectively. On REDS-II, SaMi-Trop 3, and ELSA-Brasil, the ensemble consistently outperforms both ECGFounder and the baseline model, demonstrating improved robustness and generalization across datasets despite the increased difficulty and domain shift observed in ELSA-Brasil.

ECGFounder generally achieves intermediate performance, with AUROC values of 0.722, 0.732, and 0.587 on REDS-II, SaMi-Trop 3, and ELSA-Brasil, respectively. The baseline model achieves AUROC values of 0.624, 0.588, and 0.595 across the same datasets. These results suggest that the ensemble approach more effectively aggregates complementary information from individual models, leading to improved robustness, generalization, and predictive performance across diverse datasets.

The inset panels highlight the clinically relevant low FPR region (FPR $\,{\unicode{x2A7D}}\, 0.1$). In this operating range, the ensemble achieves the highest TPR@5% on REDS-II and SaMi-Trop 3, with values of 0.389 and 0.351, respectively, compared with ECGFounder (0.314 and 0.283) and the baseline model (0.114 and 0.089). On ELSA-Brasil, TPR@5% values are lower overall and more similar across methods, with the ensemble, ECGFounder, and baseline model achieving 0.146, 0.129, and 0.118, respectively. These findings demonstrate that the ensemble approach provides the greatest improvement in clinically relevant operating regions for REDS-II and SaMi-Trop 3.

This pattern is further supported by the TPR@5% results summarized in table 5. The baseline model achieves relatively low sensitivity across all three datasets (0.114 on REDS-II, 0.089 on SaMi-Trop 3, and 0.118 on ELSA-Brasil), with an average score of 0.107. In contrast, all ensemble approaches substantially improve upon this baseline. Among the ensemble methods, the arithmetic mean ensemble achieves the strongest overall performance, with TPR@5% values of 0.389 on REDS-II, 0.351 on SaMi-Trop 3, and an average score of 0.295.

**Table 5.** Challenge score, TPR@5%, for different models under each individual test dataset. The baseline and foundation model were developed independently prior to the Challenge and were not included in the ensemble models.

Model/Team	REDS-II	SaMi-Trop 3	ELSA-Brasil	Average
Baseline model (Jidling *et al* 2023)	0.114	0.089	0.118	0.107
Foundation model: ECGFounder (Li *et al* 2025a)	0.314	0.283	0.129	0.242

Biomed-Cardio (19) (Van Santvliet *et al* 2025)	**0.468**	**0.376**	0.125	**0.323**
DlaskaLabMUI (236) (Nicolson *et al* 2025)	0.357	0.375	0.118	0.283
AIChagas (100) (González Vázquez *et al* 2025)	0.382	0.329	0.129	0.280
ISIBrno-AIMT (132) (Nejedly *et al* 2025)	0.354	0.342	0.118	0.271
Ahus AIM (298) (Stenhede and Ranjbar 2025)	0.389	0.325	0.093	0.269
cardio_Basel25 (42) (Champetier and Mueller 2025)	0.289	0.355	0.125	0.256
CinCo Amigos (158) (Wang *et al* 2025b)	0.339	0.247	**0.164**	0.250
Chagas_detector (416) (Nguyen *et al* 2025)	0.329	0.281	0.125	0.245
AIMED (368) (Soares *et al* 2025)	0.321	0.295	0.104	0.240
Chagas_UBT (126) (Mamaghani *et al* 2025)	0.314	0.295	0.104	0.238

Ensemble: Arithmetic mean	0.389	0.351	0.146	0.295
Ensemble: Geometric mean	0.378	0.354	0.136	0.289
Ensemble: Median	0.371	0.342	0.136	0.283

The ECGFounder foundation model achieves intermediate performance, with TPR@5% values of 0.314, 0.283, and 0.129 across REDS-II, SaMi-Trop 3, and ELSA-Brasil, respectively, corresponding to an average score of 0.242. While ECGFounder outperforms the baseline model and several individual challenge models, the ensemble approaches consistently achieve higher sensitivity on REDS-II and SaMi-Trop 3.

Although the ensemble approaches did not outperform the strongest individual Challenge model in terms of average Challenge score, they exhibited lower performance variability across the external test datasets. Specifically, while the top-performing individual model achieved higher scores on REDS-II and SaMi-Trop 3, its performance decreased substantially on ELSA-Brasil, whereas the ensemble approaches showed more consistent performance across the three datasets. These findings suggest that ensemble aggregation may provide greater robustness to dataset heterogeneity, although this may come at the expense of achieving the highest performance on individual datasets or in terms of the overall average Challenge score.

## 4. Discussion

The differences in model performance between the different data sources in the test set reflect the difficulty of generalizing to unseen data. While the models performed similarly on the REDS-II data in the validation and test sets, they performed moderately worse on the SaMi-Trop 3 data, which has a similar patient population to the REDS-II data, and much worse on the ELSA-Brasil data, which had a largely asymptomatic patient population.

Notably, while the models performed worst on the ELSA-Brazil data, this dataset best mirrors potential data for a national ECG-based screening campaign for Chagas disease in Brazil. Even then, the highest-performing models identified nearly three times as many Chagas-positive patients as indiscriminate testing, potentially arresting Chagas disease development in patients while they remain largely asymptomatic.

Relatedly, Chagas disease prevalence rates and serological testing capacities vary geographically and evolve over time. The prevalence rate of 2% and the serological testing capacity of 5% is roughly accurate for Brazil at this time l, but interventions to reduce the transmission of Chagas disease may reduce the rates even further. To help automated approaches ECG-based screening to generalize beyond the training set, careful consideration about population matching and domain shifts are needed.

Our observations about the Challenge models provide insights for future improvements. First, the teams generally did not account for non-causal differences between databases, and they generally did not distinguish between strong and weak labels in the training data. Very few teams explicitly addressed these issues. Second, despite the large body of evidence demonstrating an association between HRV and Chagas disease (Cardoso *et al* 2015, Vizcardo and Ravelo Garcia 2018, Vizcardo *et al* 2019, Rodrigo Cornejo Luque *et al* 2020, Cornejo *et al* 2022, Isaac Valdez *et al* 2023, Rodriguez *et al* 2023), none of the top-performing PhysioNet Challenge 2025 approaches explicitly incorporated traditional HRV metrics into their prediction pipelines. Instead, the highest-ranked teams primarily relied on deep representation learning directly from raw ECG waveforms using convolutional neural networks, transformers, ECG foundation models, self-supervised learning, and ensemble methods. While these architectures may implicitly capture certain rhythm-related characteristics, it is unlikely that subtle beat-to-beat variability patterns were robustly learned from the relatively short ECG segments available for analysis. This observation is consistent with the modest performance previously reported for HRV-only prediction algorithms (Silva *et al* 2022), suggesting that HRV alone may not provide sufficient discriminative power for Chagas disease detection, but it could still represent a complementary and under explored source of physiological information when combined with morphology-based ECG representations.

Similarly, our observations about other models on the Challenge task provide insights about diagnosing Chagas disease from ECGs. The voting-based ensemble strategy, which aggregates predictions from multiple models using arithmetic mean, median, and geometric mean, demonstrated a consistent and robust improvement over individual models. By integrating complementary information across the top-performing Challenge models, this approach reduced prediction variability and enhanced overall discriminative performance relative to both individual models and the baseline model (Jidling *et al* 2023). Among all ensemble strategies, the arithmetic mean ensemble exhibited the most stable performance, suggesting an improved ability to balance contributions across models while attenuating the influence of extreme predictions.

Interestingly, the ECGFounder foundation model also demonstrated competitive performance across datasets, generally achieving results within the range of the top-performing individual Challenge models and substantially outperforming the baseline model on REDS-I, SaMi-Trop 3 and ELSA-Brasil. ECGFounder was included in our comparative analysis as an ECG-based foundation reference model. Specifically, while the baseline model provides a standard reference point for the Challenge task, ECGFounder represents a more sophisticated foundation-model-based approach that leverages large-scale pretraining on diverse ECG data. Its inclusion therefore allowed us to assess whether the performance gains observed with the ensemble approaches persisted when compared against a substantially stronger standalone reference model. This finding suggests that large-scale pretraining on diverse ECG data can provide robust and transferable feature representations for Chagas disease screening. However, despite its strong standalone performance, ECGFounder was consistently outperformed by the ensemble approaches, particularly in clinically relevant low FPR operating regions. Thus, the advantage of the ensemble approaches was not limited to comparisons with the conventional baseline but was also observed relative to a more advanced foundation model. This observation suggests that ensemble aggregation may provide additional robustness and generalization benefits across external cohorts beyond those achieved by a single foundation model.

The benefits of the ensemble approach were particularly evident at clinically relevant operating points constrained by screening capacity. Under these conditions, ensemble models achieved higher sensitivity than individual and foundation models without a commensurate increase in FPR, indicating a more efficient prioritization of high-risk individuals for downstream evaluation. This finding underscores the capacity of ensemble methods to capture complementary and heterogeneous signal characteristics that may not be reliably detected by any single model.

Feature importance analyses from the ECGFounder embeddings and SHAP-based interpretation consistently suggest that conduction-system and ventricular depolarization abnormalities are among the most predictive ECG signatures of Chagas disease (figure S1a, S1b, and S1c from the Supplementary Material). Across the REDS-II, SaMi-Trop 3, and ELSA-Brasil datasets, several ECG abnormalities repeatedly emerged among the highest-ranked and most reproducible SHAP features, indicating that ECGFounder learned stable electrophysiological signatures of chronic Chagas cardiomyopathy across heterogeneous populations. The most consistently important features included electronic ventricular pacemaker patterns, marked sinus bradycardia, ventricular escape complexes, left anterior fascicular block, wide QRS rhythms, bifascicular block, prolonged QT intervals, abnormal left axis deviation, and nonspecific T-wave abnormalities. Collectively, these findings primarily reflect conduction-system disease, bradyarrhythmias and escape rhythms, ventricular depolarization abnormalities, fascicular and bundle branch blocks, and repolarization abnormalities characteristic of chronic Chagas cardiomyopathy. Importantly, the cross-dataset rank consistency analysis (figure S1a, S1b, and S1c from the Supplementary Material) demonstrated that many of these ECG abnormalities remained highly ranked across all three datasets rather than being driven by a single cohort-specific effect. Features such as electronic ventricular pacemaker patterns, marked sinus bradycardia, ventricular escape complexes, left anterior fascicular block, and conduction-related QRS abnormalities consistently demonstrated low mean feature ranks and tight clustering across datasets, suggesting strong reproducibility of their predictive contribution. In contrast, several lower-ranked features exhibited greater variability across datasets, indicating more dataset-specific importance.

These ECGFounder-derived findings showed strong conceptual agreement with the dominant ECG themes emphasized across the top-performing PhysioNet Challenge 2025 models. Although the Challenge approaches differed substantially in architecture and training strategy, many converged on electrophysiological patterns associated with conduction-system abnormalities, arrhythmias, ventricular ectopy, bundle branch and fascicular blocks, QRS morphology, and broader ECG waveform representations as important indicators of Chagas disease. Several top-performing teams also highlighted the importance of physiologically meaningful intervals and morphology-based representations related to *P*–*R* intervals, QRS complexes, atrioventricular conduction, and rhythm abnormalities. The overlap between these independently developed Challenge approaches and the ECGFounder SHAP interpretations suggests that the foundation model consistently captured clinically meaningful and reproducible electrophysiological signatures of chronic Chagas cardiomyopathy across heterogeneous datasets. At the same time, ECG-based models also captured Chagas disease patients with normal ECGs, although with lower performance.

An important observation from the hidden external evaluation was that ensemble-based prediction strategies demonstrated greater stability and robustness on the fully unseen ELSA-Brasil dataset. Although several individual models achieved strong performance on development datasets, their reduced performance on external hidden data suggests varying degrees of overfitting. In contrast, combining complementary model predictions improved generalization across datasets and provided more consistent performance in unseen populations. These findings highlight the potential importance of ensemble approaches for improving robustness, reducing overfitting, and enhancing the real-world applicability of ECG-based Chagas disease screening systems.

## 5. Conclusions

This article describes a large compendium of public and private 12-lead ECGs from several sources with self-reported and serologically validated Chagas disease status.

The combination of standard 12-lead ECGs with a large database with weak labels and several smaller databases with strong labels poses a classical machine learning problem in a real-world setting, and the use of three completely hidden data sources with varying patient populations and environments helps to assess model generalizability to unseen data.

Moreover, the use of an evaluation metric that specifically incorporates the confirmatory testing capacity for Chagas disease helps to support the development of clinically relevant machine learning models for detecting Chagas disease as part of an effort to prioritize testing.

The performance of the Challenge algorithms varied across databases, but the top-performing algorithms were able to identify a large fraction of Chagas patients, ranging from one in two Chagas patients in a cohort of largely symptomatic patients to one in six Chagas patients of largely asymptomatic patients, outperforming the expected rate of one in fifty for a random model.

Despite these limitations, the 2025 George B. Moody Challenge pushed forward the field of Chagas disease diagnostics with the ECG beyond 24 h Holter monitoring, and provided promising directions for introducing a clinical Chagas screening system based on short ECG recordings at the point-of-care.

## Data Availability

The data that support the findings of this study are openly available at the following URL/DOI: https://physionetchallenges.org/2025/. Supplementary data 1 available at https://doi.org/10.1088/1361-6579/aead44/data1.
